# DPP4 Activities Are Associated with Osteopenia/Osteoporosis and Fracture Risk in Newly Diagnosed Type 2 Diabetes

**DOI:** 10.1155/2020/8874272

**Published:** 2020-11-27

**Authors:** Min Qiu, Shuheng Zhai, Da Liu

**Affiliations:** ^1^Department of Orthopedics, Shengjing Hospital of China Medical University, Shenyang, Liaoning, China; ^2^Department of Obstetrics and Gynecology, Shengjing Hospital of China Medical University, Shenyang, Liaoning, China

## Abstract

**Background:**

Recent studies have shown the beneficial effect of dipeptidyl peptidase-4 (DPP4) inhibitor on bone turnover in diabetes mellitus. However, little clinical evidence for DPP4 activity in newly diagnosed type 2 diabetes is available. This study was designed to investigate the relationship between plasma DPP4 activity and osteoporosis/osteopenia and fracture risk in newly diagnosed type 2 diabetes.

**Methods:**

A total of 147 subjects with newly diagnosed type 2 diabetes were enrolled for this cross-sectional study. The bone mineral density (BMD) at the lumbar spine (L1-4) and femoral neck (FN) was measured by dual-energy X-ray absorptiometry (DXA). The 10-year probability of major osteoporotic fracture (MOF) and hip fracture (HF) was assessed by a modified fracture risk algorithm (FRAX) tool. The plasma DPP4 activity and clinical variables were measured. Correlation analyses between DPP4 activity and osteoporosis/osteopenia and fracture risk were performed.

**Results:**

Elevated plasma DPP activities were significantly associated with a higher proportion of osteoporosis/osteopenia (50% for quartile-1, 56.4% for quartile-2, 65.8% for quartile-3 and 72.2% for quartile-4). With increasing plasma DPP activities, the incidence rate of osteoporosis/osteopenia is gradually increasing (*P* for the trend between quartiles = 0.04). Of note, a statistically significant linear correlation was found between plasma DPP4 activities and modified FRAX MOF (*r* = 0.20, *P*=0.02). Moreover, plasma DPP4 activities were also positively related to modified FRAX HF in newly diagnosed type 2 diabetic patients (*r* = 0.21, *P*=0.01).

**Conclusions:**

Elevated plasma DPP4 activity tended to be associated with a higher proportion of osteoporosis/osteopenia and increased the fracture risk in newly diagnosed type 2 diabetes.

## 1. Background

The prevalence of diabetes mellitus is increasing worldwide with diabetes-related complications, imposing a tremendous burden on all health-care systems [1]. Of note, fragility fractures are increasingly recognized as an important complication of diabetes mellitus and are associated with substantial morbidity and mortality [2]. Diabetic osteopathy may experience various musculoskeletal disorders, such as osteoporosis, osteopenia, and diabetic foot syndrome, which is an underlying condition characterized by microarchitectural changes that can reduce bone quality and increase the risk of bone fractures [3]. Osteoporosis/osteopenia is the most common metabolic disorder in the bone characterized by decreasing the density of normally mineralized bone. Although evidence from both the bench and the bedside has shown a strong interaction between glucose homeostasis and bone metabolism, the mechanisms underlying the detrimental effects of diabetes on skeletal health remain not clearly defined [4].

Poor diabetes management can have adverse consequences such as heart disease, renal failure, osteoporosis, and even death, which are potentially preventable by optimal metabolic control. Antidiabetic drugs are very important for glycemic control in all diabetic patients; however, they may increase osteoporosis and fracture risk in diabetes mellitus [5]. In contrast, it has been reported that recently marketed antidiabetic drugs including incretins and dipeptidyl peptidase-4 (DPP4) inhibitors can potentially improve bone quality [6]. DPP4, also known as CD26, is a crucial factor in the regulation of insulin secretion and glucose homeostasis. Notably, there is growing evidence that DPP4 may have an important role in bone formation, bone resorption, and bone microstructure [6, 7]. The recent meta-analysis of randomized clinical trials also suggests that treatment with DPP4 inhibitors could be associated with a reduced risk of bone fractures in type 2 diabetes [7]. Besides, previous studies have shown that the beneficial effect of glucagon-like peptide-1 has an in vivo half-life of only a couple of minutes because of rapid inactivation by DPP4, on the bone formation or resorption [8]. Preliminary data on animals and preclinical studies suggest the hypothesis that DPP4 inhibitors could have a positive effect on bone metabolism by a direct effect on bone cells; however, clinical studies are needed to elucidate the association of DPP4 activity with bone metabolism [9].

The fractures risk algorithm (FRAX) is an online tool widely used by many professional institutions for fracture risk assessment [10]. There is evidence showing that diabetes is recommended to replace rheumatoid arthritis in FRAX to effectively improve FRAX performance in diabetic patients [11–13]. The modified FRAX employs clinical risk factors such as age, body mass index (BMI), previous fractures, and other factors combined with femoral neck (FN) bone mineral density (BMD) determined by dual X-ray absorptiometry (DXA) to estimate the 10-year probability of major osteoporotic fracture (MOF) and hip fracture (HF) [13].

Despite knowledge of the importance of DPP4, little is known about the relation between circulating DPP4 activities and diabetic osteopathy in newly diagnosed type 2 diabetic patients. Accordingly, we set out to explore the associations of plasma DPP4 activities with osteoporosis/osteopenia and the ten-year probability of major osteoporotic fracture (MOF) and hip fracture (HF) estimated with modified FRAX in new onset type 2 diabetic patients.

## 2. Materials and Methods

### 2.1. Study Population

To explore the association between plasma DPP4 activities and osteoporosis/osteopenia and the ten-year probability of major osteoporotic fracture (MOF) and hip fracture (HF) estimated with modified FRAX, 158 newly diagnosed type 2 diabetic patients were enrolled from subjects undergoing routine health checkup at Shengjing Hospital of China Medical University (Shenyang, China) from December 2017 to May 2019. All of the enrolled subjects underwent the oral glucose tolerance test (OGTT). Type 2 diabetes was diagnosed based on the American Diabetes Association guideline [14]. They had no history of taking medications such as blood pressure medications and lipid-lowering medications. Participants with gestational diabetes, cerebrovascular diseases, chronic renal diseases, and hepatic diseases were excluded from this study. None of the participants received insulin therapy or antidiabetic medication, and therefore, their blood glucose was not affected. Exclusion criteria also included the use of agents that may affect bone metabolisms, such as thiazolidinediones, vitamin K, warfarin, vitamin D, calcium supplement, bisphosphonates, and estrogen, and agents that may lower lipid levels. In this study, type 1 diabetic patients were carefully excluded from clinical grounds, based on fasting C-peptide levels and islet-associated negative autoantibodies, and from a review of medical records, All participants had signed informed written consent prior to participating in the study. The institutional review board of Shengjing Hospital has approved the present study, and all procedures were carried out following the principles expressed in the Declaration of Helsinki.

### 2.2. Collection and Definition of Clinical Variables

The medical information was collected based on the medical records. BMI was calculated as weight in kilograms divided by height in meters squared. To avoid potential confounding effects, samples of venous blood were drawn after an overnight fast. Clinical biochemical variables were determined at the Department of Medical and Chemical Laboratory Diagnostics of Shengjing Hospital according to routine procedures. DPP4 activity in plasma was assayed as previously reported [15]. Briefly, plasma DPP4 activity was determined as the rate of cleavage of 7-amino-4-methylcoumarin (AMC) from the synthetic substrate H-glycyl-prolyl-AMC (H-Gly-Pro-AMC; Biovision, San Francisco, California, USA). It is expressed as the amount of cleaved AMC per minute per ml (nmol/min/ml). The insulin resistance was evaluated by homeostasis model assessment of insulin resistance (HOMA-IR), and the beta cell function was evaluated by homeostasis model assessment of insulin secretion (HOMA-IS) as previously reported [16].

### 2.3. BMD Measurement and Fracture Risk Assessment

The areal bone mineral density (BMD) (g/cm^2^) of all participants was measured at the lumbar spine (L1–L4) and femoral neck (FN) by dual-energy X-ray absorptiometry (Lunar Prodigy). Accordingly, osteoporosis is diagnosed by a *T*-score ≦ −2.5 SD and osteopenia is diagnosed by a −1 ≧ *T*-score > −2.5 SD at any of the sites on the lumbar spine or FN [17]. The 10-year probability of fractures was determined with the modified FRAX tool (https://www.sheffeld.ac.uk/FRAX/tool.aspx?country=2), with the following parameters: age, sex, weight, height, fracture history, parental history of hip fractures, glucocorticoid usage, RA (diabetes in the present study), smoking status, and alcohol intake [11–13]. A China-specific FRAX algorithm with FN-BMD was selected to evaluate the 10-year probability of major osteoporotic fracture (MOF) and hip fracture (HF) [13].

### 2.4. Statistical Analysis

Continuous variables are presented as means ± standard deviation (SD), median (25th and 75th percentiles). Categorical variables are presented as percentage. Normal distribution of continuous variables was determined using the one-sample Kolmogorov–Smirnov test. Continuous variables with a normal distribution were assessed by one-way ANOVA with post hoc Tukey's test. Nonnormally distributed data were tested for nonparametric distribution. Correlation analysis between continuous variables was performed by Spearman's analysis. Categorical variables were examined by the chi-squared test. The Statistical Package for Social Science (SPSS) version 15.0 was applied to perform all statistical and association analyses. Two-tailed tests were adopted throughout, and *P* values less than 0.05 were considered.

## 3. Results

### 3.1. Clinical Features

A total of 147 newly diagnosed type 2 diabetic patients were evaluated. They were divided into three groups, normal bone mineral density group (*n* = 57), osteopenia group (*n* = 64), and osteoporosis group (*n* = 26), according to *T*-score by dual-energy X-ray absorptiometry. The baseline clinical characteristics of the three groups are depicted in Table 1. We observed significant differences in gender among these groups. The osteoporosis group had a significantly higher ratio of females than the osteopenia group and normal bone mineral density group (65.4% vs. 45.3% and 35.1%, respectively; *P*=0.02). As expected, compared with the normal bone mineral density group and osteopenia group, the average age in the osteoporosis group was significantly increased (53.4 ± 3.2, 55.7 ± 4.1, and 57.6 ± 5.0, respectively; *P* < 0.01). Of note, plasma DPP4 activity tended to be marginally higher in the osteoporosis group compared with the osteopenia group and normal bone mineral density group (7.7 ± 0.8 vs. 7.5 ± 0.9 and 7.3 ± 0.8 nmol/min/ml, respectively; *P*=0.07). There were no statistically significant differences in other clinical characteristics among these groups (all *P* > 0.05).

### 3.2. Correlations between DPP4 Activity and Osteoporosis/Osteopenia

**Figure 1:**
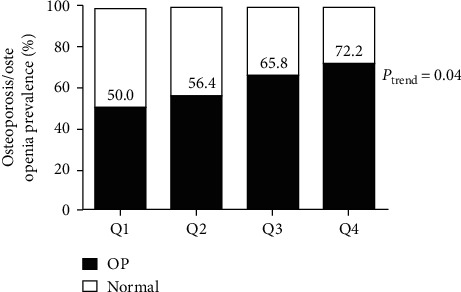
Prevalence rate of osteoporosis/osteopenia according to plasma DPP4 activity quartiles. Q1: <6.78 (nmol/min/ml), Q2: 6.78–7.39 (nmol/min/ml), Q3: 7.40–8.06 (nmol/min/ml), and Q4: >8.06 (nmol/min/ml). Normal, normal bone mineral density (*T*-score ≥ −1); OP, osteoporosis/osteopenia (*T*-score < −1) (linear-by-linear association for the trend test).

To achieve an even distribution in each group, the subjects were divided into subgroups using DPP4 activity quartiles: Q1: <6.78 (nmol/min/ml); Q2: 6.78–7.39 (nmol/min/ml); Q3: 7.40–8.06 (nmol/min/ml); Q4: >8.06 (nmol/min/ml). Although there was a positive correlation between DPP4 activity and the prevalence of osteoporosis, the trends were not statistically significant (*P* > 0.05). However, higher DPP activities were significantly associated with a higher proportion of osteoporosis/osteopenia in newly diagnosed type 2 diabetic patients (50% for Q1, 56.4% for Q2, 65.8% for Q3, and 72.2% for Q4) (Table 2). The prevalence of osteoporosis/osteopenia showed an increasing trend with the increase in plasma DPP4 activity (*P* for the trend between quartiles = 0.04) (Figure 1).

### 3.3. Correlations among DPP4 Activity and 10-Year Probability of MOF and HF

**Figure 2:**
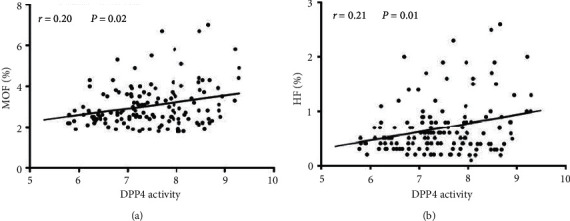
Correlations of plasma DPP4 activity with the 10-year probability of MOF (a) and HF (b) in all participants (calculated by Spearman's correlation analysis).

Spearman correlation analysis was used to determine the relationship between plasma DPP4 activity and the 10-year probability of MOF and HF. We also studied correlations between DPP4 activity and clinical variables. As shown in Table 3, plasma DPP4 activities were marginally positively correlated with HbA1c in all subjects (*r* = 0.17, *P*=0.04). In contrast, no significant correlation was found between plasma DPP4 activities and clinical parameters in newly diagnosed type 2 diabetic patients (all *P* > 0.05). Of note, a marginal linear correlation was found between plasma DPP4 activities and modified FRAX MOF (*r* = 0.20, *P*=0.02) (Figure 2). Furthermore, plasma DPP4 activities were also positively related to modified FRAX HF in all subjects (*r* = 0.21, *P*=0.01) (Figure 2).

## 4. Discussion

The present study cross-sectionally examined the relationship of plasma DPP4 activities to osteoporosis/osteopenia and fracture risk in newly diagnosed type 2 diabetes. Evidence exists in the literature that DPP4 has been identified as a novel protease playing crucial roles in the development of dyslipidemia, inflammation, and insulin resistance, all of which have been suggested to be involved in the pathogenesis of osteoporosis [18]. Our findings extend these observations by demonstrating that elevated plasma DPP4 activities were closely associated with a higher proportion of osteoporosis/osteopenia in newly diagnosed type 2 diabetic patients. Furthermore, our data for the first time indicate that plasma DPP4 activities were also positively related to the 10-year probability of major osteoporotic fracture and hip fracture estimated by modified FRAX in newly diagnosed type 2 diabetic patients.

The findings are consistent with previous data [19] and also indicate that plasma DPP4 activities associate positively with HbA1c. Chronic hyperglycemia may lead to the activation of DPP4, and long-term exposure to high glucose levels may lead to endothelial damage with a consequent increase in DPP4 secretion. In a prospective study from Italy, variations in DPP4 activity over 3 months in type 2 diabetic patients showed a significant positive correlation with variations in HbA1c [20]. Although its overall significance for the normal physiological regulation of glucose homeostasis in humans and its role in the pathogenesis of the metabolic disease remain to be established, it is evident that DPP4 has the potential to influence glycemic control [21].

Although obesity is being an important risk factor for type 2 diabetes and DPP4 being a protease, the association between circulating DPP4 activity and obesity remains debatable. However, no significant correlation was found between circulating DPP4 activity and BMI in our study. This is in line with results from a previous study, which found that adipose tissue-derived DPP4 does not significantly contribute to the active pool of plasma DPP4 activity [22]. It is noteworthy that plasma DPP4 enzyme activity was shown to be positively correlated with BMI in young healthy Japanese subjects [23]. The explanation for the existence of contradictory results lies largely in the dynamic plasma DPP4 activity from adolescence to adulthood [24].

The discovery of the incretins opens up a novel therapy in the treatment of diabetes. Incretins are gut-derived hormones that exert their actions through activation of incretin receptor signaling. In addition to its well-known glycemic control and cardioprotective effects [25, 26], it has also been identified as a novel protease playing crucial roles in bone metabolism [8]. DPP4 is a widely expressed multifunctional serine peptidase that exists as a membrane-anchored cell surface protein or in a soluble form in the plasma and degrades incretin hormones to inactive metabolites [21]. Bone cells, including osteoblasts and osteoclasts, have been shown to express receptors for incretins. Many studies indicate that glucagon-like peptide (GLP) can act as an antiresorptive and anabolic hormone [27]. Furthermore, the GLP-1 receptor is essential for the control of bone resorption as mice deficient in GLP-1 receptor present with cortical porosity as a result of increased osteoclastic bone resorption activity [5]. These experimental studies indicate that incretins have a beneficial effect on bone mass and protective effects on bone quality. As the pharmacological effect of DPP4 inhibitors is to prolong the action of GLP-1, their effect on bone is assumed to be similar to that of GLP-1. Thus, the DPP4 inhibitor seems to have an anabolic effect on bone, attenuating bone loss and potentially reducing fracture risk in type 2 diabetic patients. However, clinical data on the association between DPP4 and human bone are limited.

The present study showed for the first time that high plasma DPP4 activity was associated with the prevalence of osteoporosis/osteopenia in newly diagnosed type 2 diabetic patients (*P*_trend_=0.04). Similarly, the previous study showed a positive correlation between plasma DPP4 activity and osteoporosis in postmenopausal women with normal glucose tolerance [18]. Furthermore, our results revealed that plasma DPP4 activity was also positively related to fracture risk determined with modified FRAX in newly diagnosed type 2 diabetic patients. In line with this, the previous meta-analysis suggests that treatment with DPP4 inhibitors could be associated with a reduced risk of bone fractures [7].

Accumulating clinical evidence has demonstrated that plasma DPP4 activity is significantly increased in human subjects with polycystic ovary syndrome and metabolic syndrome [28]. Moreover, plasma DPP4 activity is reported to increase in individuals with type 2 diabetes and associate with signs of endothelial dysfunction such as impaired flow-mediated dilatation [29]. The previous study also showed that excessive activity of plasma DPP4 is independently associated with subclinical left ventricular systolic and/or diastolic dysfunction in type 2 diabetes [30]. Interestingly, there is evidence showing that increased DPP4 activity is associated with a high risk of mild cognitive impairment in elderly type 2 diabetes [31]. These evidences indicate that DPP4 has become an important molecule associated with a variety of diseases. At present, the underlying molecular mechanism how elevated plasma DPP4 activity is involved in diabetic bone and fractures in new onset type 2 diabetic patients has not been clear yet. Therefore, future studies should be performed to elucidate the function of DPP4 in the pathogenesis of bone metabolism in diabetes mellitus.

Several limitations of this study should also be considered. The incidence of osteoporosis/osteopenia and bone fractures is closely related to the hormones in postmenopausal women. It was not to discriminate between genders and between premenopausal and postmenopausal women in the present study. Furthermore, several confounders, such as daily dietary calcium intake and consumption of vitamin D, were difficult to be obtained in this epidemiological study. Osteoporosis/osteopenia was also found to be significantly associated with body mass index. Of note, most of the subjects were lean mass in this study. Finally, the present study fails to address the precise role of DPP4 in the pathogenesis of osteoporosis/osteopenia which is needed to be elucidated by the future investigation.

## 5. Conclusion

The present study revealed that elevated plasma DPP4 activity tended to be significantly associated with osteoporosis/osteopenia and the fracture risk in newly diagnosed type 2 diabetic patients. Even though the biological mechanism has not been clear yet, the current findings provide a clue that elevated plasma DPP4 activity could suggest osteoporosis/osteopenia risk and future fracture risk in new onset type 2 diabetes.

## Figures and Tables

**Table 1:** Comparison of baseline characteristics by bone status across cohorts with newly diagnosed type 2 diabetes.

Variables	Normal (*n* = 57)	Osteopenia (*n* = 64)	Osteoporosis (*n* = 26)	*P* value
Female, *n* (%)	20 (35.1)	29 (45.3)	17 (65.4)	0.02
Smoking, *n* (%)	20 (35.1)	27 (42.2)	7 (26.9)	0.38
Age (years)	53.4 ± 3.2	55.7 ± 4.1	57.6 ± 5.0	<0.01
BMI (kg/m^2^)	25.3 ± 2.1	25.0 ± 1.7	24.9 ± 1.7	0.66
HbA1c (%)	6.5 ± 0.4	6.6 ± 0.4	6.6 ± 0.4	0.85
FBG (mmol/L)	8.2 ± 1.4	8.2 ± 1.4	8.1 ± 1.1	0.96
HOMA-IR	5.4 ± 1.6	5.4 ± 1.2	5.4 ± 1.1	0.98
HOMA-IS	69.2 ± 25.0	71.8 ± 30.7	71.7 ± 23.3	0.86
TG (mmol/L)	1.6 ± 0.7	1.7 ± 1.0	1.6 ± 0.7	0.75
TC (mmol/L)	4.7 ± 1.2	5.0 ± 1.1	4.7 ± 1.1	0.42
LDL (mmol/L)	2.8 ± 0.7	2.8 ± 0.6	2.6 ± 0.7	0.51
HDL (mmol/L)	1.3 ± 0.3	1.3 ± 0.3	1.3 ± 0.3	0.83
BUN (mmol/L)	5.1 ± 1.5	5.1 ± 1.4	5.4 ± 1.9	0.71
Cr (*μ*mol/L)	73 ± 23	76 ± 20	70 ± 18	0.44
UA (*μ*mol/L)	409 ± 80	409 ± 78	416 ± 85	0.90
DPP4 activity (nmol/min/ml)	7.3 ± 0.8	7.5 ± 0.9	7.7 ± 0.8	0.07

Data are presented as mean ± SD or percentages. BMI, body mass index; HbA1c: hemoglobin A1c; FBG: fasting blood glucose; HOMA-IR: homeostatic model assessment of insulin resistance; HOMA-IS: homeostasis model assessment of insulin secretion.

**Table 2:** Prevalence rate of osteoporosis/osteopenia according to plasma DPP4 activity quartiles.

	Q1 (*n* = 34)	Q2 (*n* = 39)	Q3 (*n* = 38)	Q4 (*n* = 36)	*P*_trend_
DPP4 activity (nmol/min/ml)	<6.78	6.78–7.39	7.40–8.06	>8.06	—
Osteoporosis, *n* (%)	4 (11.8)	7 (17.9)	7 (18.4)	8 (22)	0.28
Osteoporosis/osteopenia, *n* (%)	17 (50.0)	22 (56.4)	25 (65.8)	26 (72.2)	0.04

**Table 3:** Correlations of plasma DPP4 activity and modified FRAX and other clinical parameters in newly diagnosed type 2 diabetes.

Variables	*r*	*P*
Age (years)	0.14	0.08
BMI (kg/m^2^)	−0.06	0.45
HbA1c (%)	0.17	0.04
FBG (mmol/L)	0.08	0.35
HOMA-IR	0.15	0.08
HOMA-IS	0.03	0.70
BUN (mmol/L)	0.05	0.56
Cr (*μ*mol/L)	−0.03	0.74
UA (*μ*mol/L)	0.03	0.70
MOF (%)	0.20	0.02
HF (%)	0.21	0.01

BMI, body mass index; HbA1c: hemoglobin A1c; FBG: fasting blood glucose; HOMA-IR: homeostatic model assessment of insulin resistance; HOMA-IS: homeostasis model assessment of insulin secretion; MOF: the 10-year probability of major osteoporotic fracture; HF: the 10-year probability of hip fracture; FRAX: fracture risk algorithm.

## Data Availability

The datasets generated for this study are available upon request to the corresponding author.
